# Astounding Scientific Discovery

**Published:** 1845-08

**Authors:** 


### ASTOUNDING SCIENTIFIC DISCOVERY.

We were about to notice this wonderful discovery, but the following article, which we find in the Louisville Daily Journal, is done to our hands so well, that we must gratify ourselves by copying it.

"There are but few persons who have not been very sick at some period of their lives. All such persons have quite a vivid recollection of the pangs they then suffered. They felt vastly uncomfortable, but did not accurately understand what it was that brought about the afflictions of body and mind they experienced. We have the pleasure of announcing that philosophy, analysis, and the microscope have discovered the reason why when a person is diseased, he feels prodigiously restless and uneasy. From the New York Mirror, we learn that a very acute genius, Professor Bronson, has informed the world all about it: Listen:

"'Professor Bronson stated in his concluding lecture, last week, that if a drop of human blood be subjected to examination by the oxhydrogen microscope, and magnified some twenty millions of times, all the species of animals now existing on the earth, or that have existed during the different stages of creation for millions of years past, will there be discovered. In the blood of a healthy person, all the animalculæ are quiet and peaceable; but in the blood of a diseased person, they are furious, raging, and preying upon each other. This he stated in illustration of his position that man contains within himself all the principles of the universe. It was also asserted that if a dead cat be thrown into a pool of stagnant water, and allowed to dissolve there, a drop of water taken from any part of the pool, and examined as above, will show every species of animal of the cat kind that has ever existed on the earth, raging and destroying one another. The bodies of all the lower animals being made up of animalculæ similar to themselves; and the body of man being compounded of all that is below in the scale of creation.'

"We, like most other specimens of humanity, have been the victims of fever, during the continuance of which, we felt as hot as fresh-baked pancakes and as uneasy as stranded eels. We were in perfect ignorance of the cause, and are thankful to the learned Professor for the valuable information he has given us.

"From the results of the Professor's profound investigations, it appears very clear that, when a man is laboring under a hot bilious fever, a most destructive warfare is going on in every drop of blood that runs in his veins. In each drop megatheriums, mammoths with awful tusks, elephants with huge trunks, lions with shaggy manes, rhinosceroses, panthers, sperm and blubber whales, spotted leopards, tigers, bald-headed eagles, hyenas, sharks, rattlesnakes, wild boars, boa constrictors, condors, snapping turtles, armadilloes, catamounts, tarantulas, antediluvian icthosaurians, scorpions, centipedes, fretful porcupines, fly-up-the-creeks, white bears, wolves, ostriches, alligators, sea serpents, mermaids, rats, cats, mice, pigs, lizards, and jackasses, together with a vast assortment of running, crawling, flying, swimming, and creeping things which existed long before a drop of human blood was created—we say that when a poor devil has a fever all these animals and many thousands more infest each drop of his blood, biting, scratching, and tearing each other in a million different and ingenious ways. No wonder a fellow feels, to speak after the fashion of sinners, devilish uncomfortable."

And no wonder that, as the jackasses abound in Bronson's blood, he is an incorrigible ass. C.

